# Trends and burden in mental disorder death in China from 2009 to 2019: a nationwide longitudinal study

**DOI:** 10.3389/fpsyt.2023.1169502

**Published:** 2023-05-12

**Authors:** Jiawen Wu, Yuzhu Wang, Lu Wang, Hengjing Wu, Jue Li, Lijuan Zhang

**Affiliations:** Clinical Center for Intelligent Rehabilitation Research, Shanghai YangZhi Rehabilitation Hospital (Shanghai Sunshine Rehabilitation Center), Tongji University School of Medicine, Tongji University, Shanghai, China

**Keywords:** mental disorders, mortality, schizophrenia, alcohol use disorder, substance use disorders

## Abstract

**Objectives:**

We aimed to elucidate trends in the crude mortality rate (CMR), age-standardized mortality rate (ASMR), and burden of mental disorders (MD) in China.

**Methods:**

A longitudinal observational study was performed using the data of MD deaths in the National Disease Surveillance System (DSPs) during 2009–2019. The mortality rates were normalized using the Segis global population. Trends in the mortality of MDs stratified by age, gender, region, and residency, respectively. The burden of MD was evaluated using age-standardized person years of life loss per 100,000 people (SPYLLs) and average years of life lost (AYLL).

**Result:**

A total of 18,178 MD deaths occurred during 2009–2019, accounting for 0.13% of total deaths, and 68.3% of MD deaths occurred in rural areas. The CMR of MD in China was 0.75/100,00 persons (ASMR: 0.62/100,000 persons). The ASMR of all MDs decreased mainly due to the decrease in ASMR in rural residents. Schizophrenia and alcohol use disorder (AUD) were the leading causes of death in MD patients. The ASMR of schizophrenia and AUD was higher in rural residents than in urban residents. The ASMR of MD was highest in the 40–64 age group. As the leading causes of MD burden, the SPYLL and AYLL of schizophrenia were 7.76 person-years and 22.30 years, respectively.

**Conclusion:**

Although the ASMR of all MDs decreased during 2009–2019, schizophrenia and AUD were still the most important causes of death for MDs. Targeted efforts focusing on men, rural residents, and the 40–64 years old population should be strengthened to decrease MD-related premature deaths.

## Introduction

The Global Burden of Disease study highlighted that mental disorders (MD) continued to be one of the significant contributors to the global burden in the past 30 years (1). Around 14% of the global burden of disease has been attributable to neuropsychiatric disorders (2). People with MD had a mortality rate more than two times higher than that of the general population (3). Over the course of the disorders, most individuals with MD frequently die from, suicide, a substance-abuse disorder, and some chronic diseases (4). People with MD often exhibit high rates of smoking, substance use, physical inactivity, and poor diet, and these negative behaviors increase risks of premature death associated with MD (4, 5).

The burden of MD in low-income and middle-income countries accounts for about three-quarters of the global MD burden. As a middle-income country, China contributes to approximately 15–20% of the global disability-adjusted life years (DALYs) attributable to mental, neurological, and substance use disorders, ranking the first in the world (6). Mental health is one of the major public health concerns in China due to the large number of patients and the significant social and economic costs. In 2009, a survey conducted in four provinces in Chinese indicated the monthly prevalence of MD among Chinese adults was approximately 17.5%, with over 173 million adults affected by one or more MDs, and an estimated 16 million adults suffered from severe mental illness (7). Results from the China Mental Health Survey conducted from 2013 to 2015 revealed that the 12-month prevalence rate of MD among Chinese adults is 9.3%, while the lifetime prevalence rate is 16.6% (8), which decreased slightly but remained at a high level. What is more important, over 90 percent of patients with severe mental illness in China have not received proper treatment (9). In 2012, depression alone accounted for 14.7% of China’s total medical expenditure (10).

China has undergone rapid economic development over the past 30 years, resulting in increased psychological stress factors and a continuous intensification of social competitiveness, and as a consequence of this development, MD has become one of the most prevalent health problems in present-day China. Given the current situation and the rapid pace of social change regarding MD, there is a pressing need to strengthen efforts toward MD prevention and safety promotion, and to urgently establish a national program for MD prevention.

This study aims to investigate the distribution and trends of deaths related to MD in China from 2009 to 2019. We also provide insights for the development of national disability-related strategies by analyzing the main causes of MD-related deaths in China. It can help aid in the prevention and management of comorbidity and reduce the incidence of non-natural deaths in this vulnerable population.

## Method

### Data resource

The raw data for this study were obtained from the Death Surveillance Data Sets compiled by the Chinese Center for Disease Control and Prevention (CDC) from the National Disease Surveillance Point system (DSPs). The DSPs system included 161 points with a population coverage of 73 million in 2004, and was expanded to cover a population of 323.8 million (24.3% of the total population) across 605 surveillance points, each covering an entire county or district in 2013 (11). Medical and health institutions at all levels and of all types are responsible units for the cause of death information report, and only medical and health personnel with the qualification of practicing physicians can fill in the medical certificate of resident death. Cases of death occurring in medical institutions are diagnosed by the treating doctor and a death certificate is completed. In case of death at home or in other places, the cause of death shall be inferred by the preventive doctor of the township health center on the basis of the deceased’s prior medical history, physical signs, and/or medical diagnosis provided by the family members or other informed individuals. According to the International Classification of Diseases, tenth Edition (ICD-10)(World Health Organization, 1992), the classification of MD is F01-F99，and we included diseases encoded within this range in the DSPs, including unidirectional depression, bipolar disorder, schizophrenia, mental disorders due to alcohol use, mental disorders due to substance use, post-traumatic stress disorder, obsessive compulsive disorder, panic disorder, insomnia, and mental developmental disorders due to lead exposure (Supplementary Table S1). We categorized the data by age groups (#0, 1 ~ 4, 5 ~ 14, 15 ~ 24, 25 ~ 34, 35 ~ 44, 45 ~ 64, 65+) (12), as well as by gender and geographic location (eastern, central, and western regions of China).

### Geographical divisions and population geography

On the basis of the first national economic census, China was also divided into three regions: east, central, and west (13). The eastern region includes 11 provincial-level administrative regions, including Beijing, Tianjin, Hebei, Liaoning, Shanghai, Jiangsu, Zhejiang, Fujian, Shandong, Guangdong, and Hainan. The central region has eight provincial-level administrative regions: Shanxi, Jilin, Heilongjiang, Anhui, Jiangxi, Henan, Hubei, and Hunan. The western region consists of 12 provincial-level administrative regions: Sichuan, Chongqing, Guizhou, Yunnan, Tibet, Shaanxi, Gansu, Qinghai, Ningxia, Xinjiang, Guangxi and Inner Mongolia. This division is a policy division, not an administrative division, nor is it a completely geographic division. Therefore, the eastern part refers to the provinces and cities that implement the coastal opening policy earlier and have a higher level of economic development. The central part refers to the less developed areas, while the western part refers to the less developed areas in the west (14, 15).

### Estimation of MD mortality and burden

To estimate the burden of MD, we calculated crude mortality rates (CMR) for 18 5-year age groups from 2009 to 2019, stratified by sex and expressed as per 100,000 persons per year. Age-standardized mortality rates (ASMR) were calculated using Segi’s world standard population (16–18). Person years of life lost (PYLL), age-standardized person years of life loss per 100,000 persons (PYLLs), and average years of life lost (AYLL) were applied to estimate the burden of mental disorders. All analyses were conducted separately for men and women and aggregated by 5-year age groups.

The formulas and calculations were as follows (19): $\mathrm{PYLL}=\sum_{i=1}^{N-1}d_{i}\left(N-i\right),$ SPYLLs = $\frac{PYLL\times 100000}{n},$ AYLL = $\frac{PYLL}{\sum d_{i}}.$ I = age at death; di = number of deaths at age *i*; *N* = upper cut-off age, 71 for men and 75 for women were adopted (20), respectively.

### Statistical analysis

Epidata 3.1 (The Epidata Association, Odense, Sydanmark, Denmark) was used for data input and SPSS 20.0 (SPSS, Inc., Chicago, IL, USA) was used for data analysis. The CMR of total MD and site-specific MD per 100,000 persons in all DSPs was calculated from 2009 to 2019. The ASMR was computed utilizing the methods recommended by Rothman and Greenland (21). We stratified the population by age, gender, region, and residency. Joinpoint Regression Software (version 4.7.0.0, provided by the Surveillance Research Program of the National Cancer Institute, Bethesda, MD) was used to analyze temporal trends in CMR, ASMR, and MD burden from 2009 to 2019. Normal test was conducted on dependent variables before use. If normal distribution was taken, Joinpoint regression prediction model was used for analysis; if the dependent variable follows Poisson or exponential distribution, log-linear model was used for analysis, and software parameter Settings was set by default. To evaluate the trend, we calculated the average annual percentage change (AAPC) and used a z-test to determine whether the APC was significantly different from zero. We used the T-test to examine differences in MD mortality rates between the two groups (by sex, urban, and rural), used the one-way analysis of variance to examine differences in MD mortality rates across regions (east, central, and west), and compared them pound-for-pair, and used Kruskal–Wallis test instead when the data did not conform to the normal distribution. Relative risk (RR) values were calculated to study the relationship between urban and rural distribution of patients with mental disorders and other factors. 95% confidence interval (CI) for each segment was calculated. *p* < 0.05 was considered as the statistically significant level.

## Results

### Basic information of MD in China, 2009–2019

**Figure 1:**
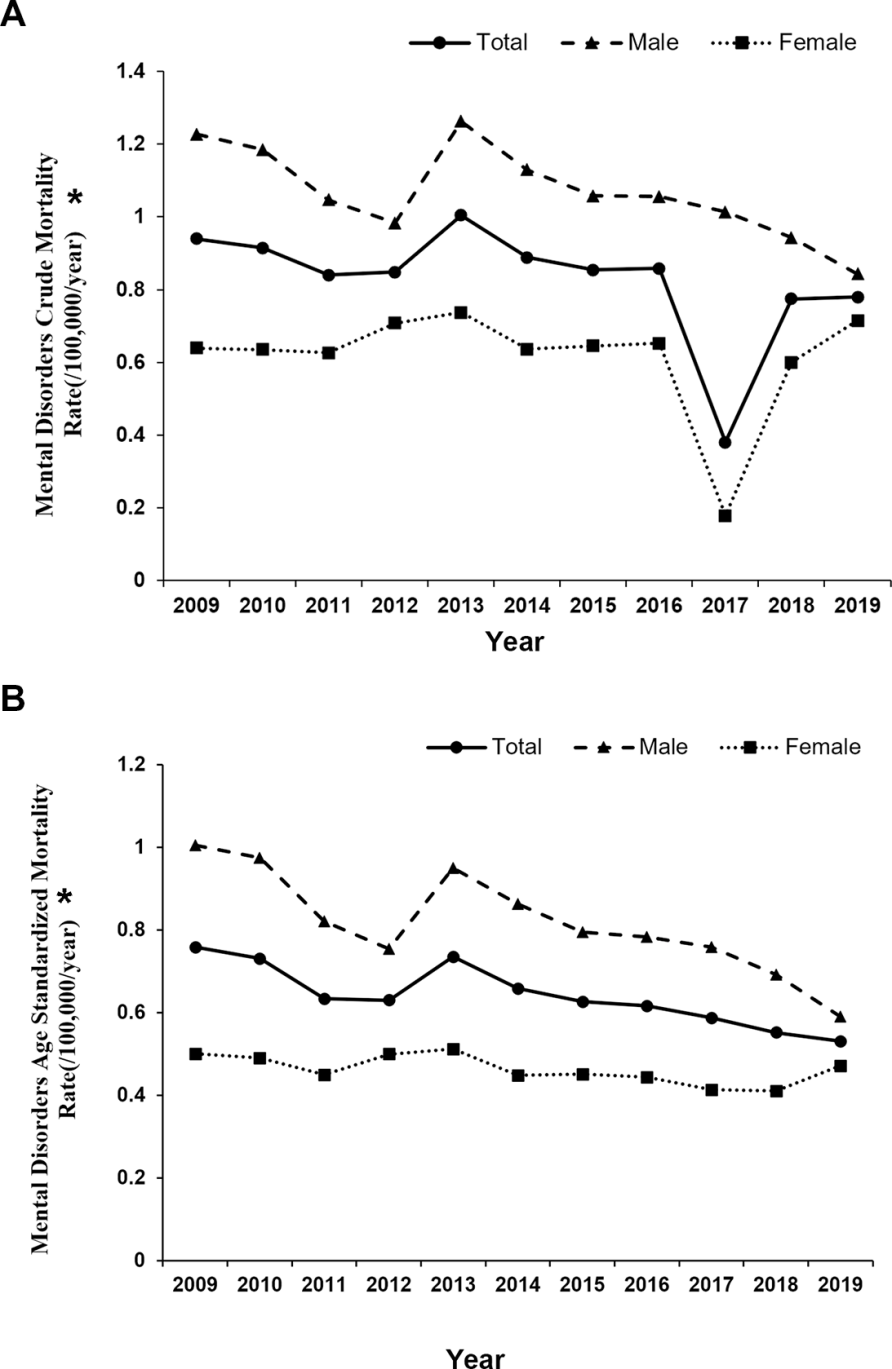
Tendency of mental disorders mortality rate among residents by gender in China, 2009–2019. Differences in ASMR between men and women: **(A)** crude mortality rate, *p* < 0.001 **(B)** age-standardized mortality rate, *p* < 0.001, * indicates *p* < 0.05.

The DSP system provides rates and proportions of MD deaths in China, classified by gender, age, and geographic region. Total 2,131,854,678 person-years were analyzed and 18,178 MD deaths (11,518 men vs. 6,660 women) occurred from 2009 to 2019, accounting for 0.13% of all-cause death. About 68.3% of MD deaths occurred in rural areas (Supplementary Table S2). During the studied period, the CMR of MD in China was 0.75 per 100,000 (1.05 per 100,000 in men and 0.51 per 100,000 persons in women) and the ASMR was 0.62 per 100,000 persons (0.78 per 100,000 men and 0.45 per 100,000 women; Supplementary Table S3). The CMR and ASMR of MD were higher in men than in women (both *p* < 0.001; Figure 1). Schizophrenia and AUD were the top two causes of MD death in the entire population (Supplementary Figure S1).

### Trends in the ASMR of the major MD

The CMR of total MD in China remained stable from 2009 to 2019, while the ASMR of total MD showed a downward trend (average annual percentage change [AAPC] −3.0, 95% CI −4.2 to −1.8), which was mainly contributed by rural residents (AAPC −5.1, 95% CI −5.7 to −4.6). The CMR of MD decreased in men (AAPC −2.6, 95% CI −4.4 to −0.8) and eastern residents (AAPC −2.2, 95% CI −3.7 to −0.7). The ASMR of MD decreased in both men (AAPC-3.9, 95% CI-5.7 to-2.0) and women (AAPC −1.5, 95% CI −2.7 to −0.2), as well as in all three regions [eastern (AAPC −2.3, 95% CI −4.2 to −0.3), central (AAPC −2.3, 95% CI −4.2 to −0.3), and western (AAPC −3.0, 95% CI −4.8 to −1.2)]. The average decrease in ASMR of MD was the highest in the group age ≥ 60 years old (AAPC −6.0, 95% CI −8.2 to −3.6), followed by the group age ≤ 39 year old (AAPC −5.5, 95% CI −8.7 to −2.2) and in the group 40–64 year old (AAPC −4.3, 95% CI −5.7 to −2.8), which was consistent with their CMR change (Supplementary Table S4).

**Figure 2:**
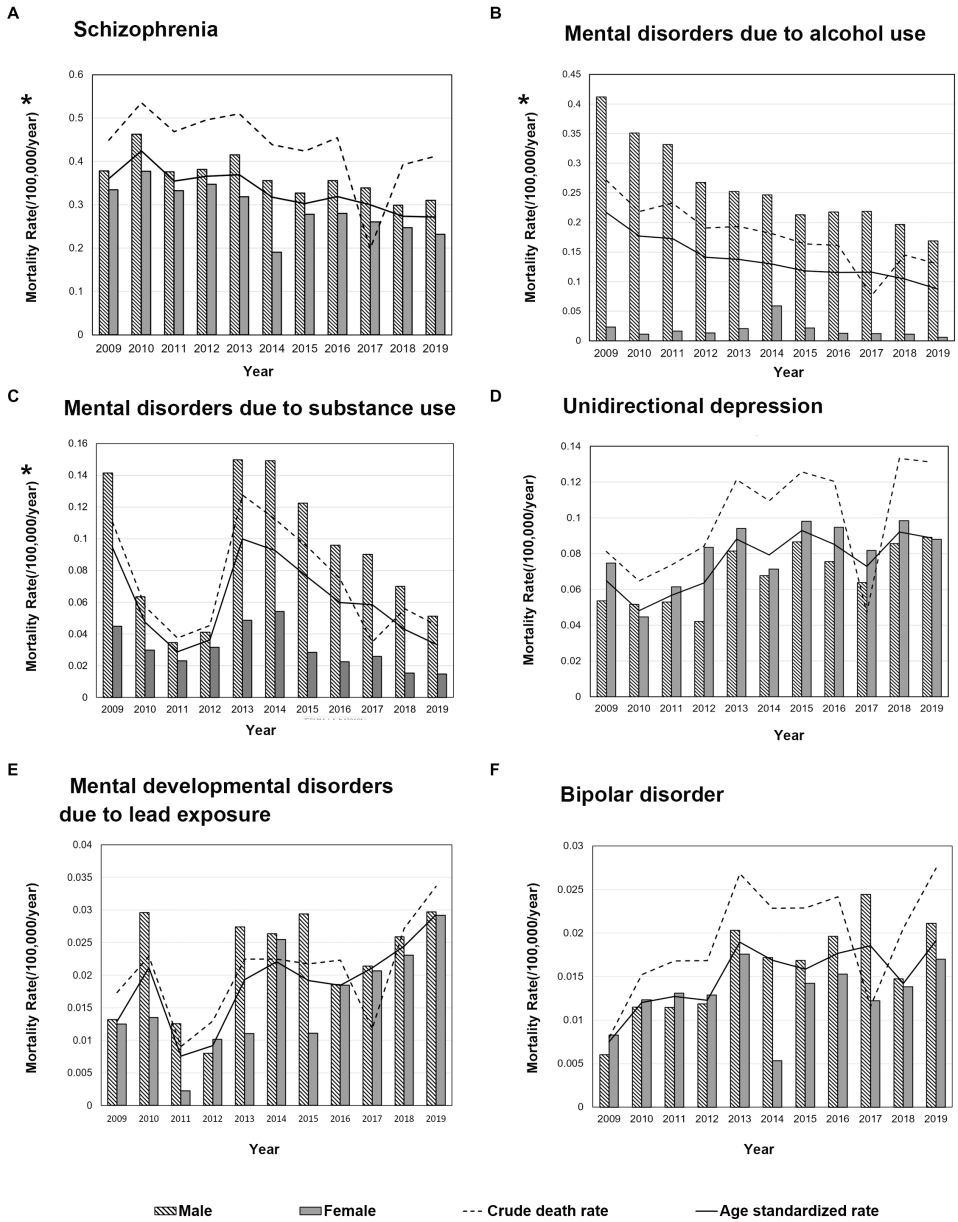
Change trends of crude and age-standardized mortality for main mental disorder in China, 2009–2019. Differences in ASMR between men and women: **(A)** Schizophrenia, *p* = 0.004 **(B)** Mental disorders due to alcohol use, *p* < 0.001 **(C)** Mental disorders due to substance use, *p* < 0.001 **(D)** Unidirectional depression, *p* = 0.088 **(E)** Mental developmental disorders due to lead exposure, *p* = 0.096 **(F)** Bipolar disorder, *p* = 0.139, * indicates *p* < 0.05.

Of the six major types of MD death, the ASMR of schizophrenia (AAPC −3.7, 95% CI −5.0 to −2.4) decreased during the 11 years, and the trend of ASMR in men (AAPC −3.1, 95% CI −4.7 to −1.4), women (AAPC −4.3, 95% CI −7.3 to −1.1) and rural areas (AAPC −5.7, 95% CI −7.6 to −3.7) was consistent with the overall population. The CMR of AUD showed a downward trend (AAPC −8.0, 95% CI −12.1 to −3.8), which was mainly contributed by men (AAPC −6.1, 95% CI −7.3 to −4.9) and rural residents (AAPC −9.9, 95% CI −15.2 to −4.3). The ASMR for AUD declined in the whole population (AAPC −7.4, 95% CI −8.7 to −6.0) and in men (AAPC −8.0, 95% CI −10.4 to −5.5), urban residents (AAPC −3.2, 95% CI −6.0 to −0.2), as well as rural residents (AAPC −9.3, 95% CI −10.7 to −7.8). The death rate of SUD in women declined in the past 11 years (CMR: AAPC −9.8, 95% CI −18.5 to 0.0; ASMR: AAPC −8.0, 95% CI −14.6 to −0.8). The CMR of unidirectional depression increased in men (AAPC 6.9, 95% CI 2.8 to 11.2), and the ASMR increased in the whole population, men, women, and urban and rural residents. The ASMR of developmental MD due to lead exposure increased at an annual average rate of 8.7 (95% CI 1.2 to 16.7, *p* = 0.026), which was mainly contributed by women (AAPC 13.8, 95% CI 0.3 to 29.1) and rural residents (AAPC 10.7, 95% CI 1.5 to 20.8). The ASMR for bipolar disorder increased at a rate of 7.3(95% CI 0.5 to 14.5) for the whole population and 9.9 for men (95% CI 3.9 to 16.3; Supplementary Table S5). The ASMRs of schizophrenia, AUD, and SUD were higher in men than in women (*p* = 0.004, *p* < 0.001, *p* < 0.001, respectively; Figure 2).

**Figure 3:**
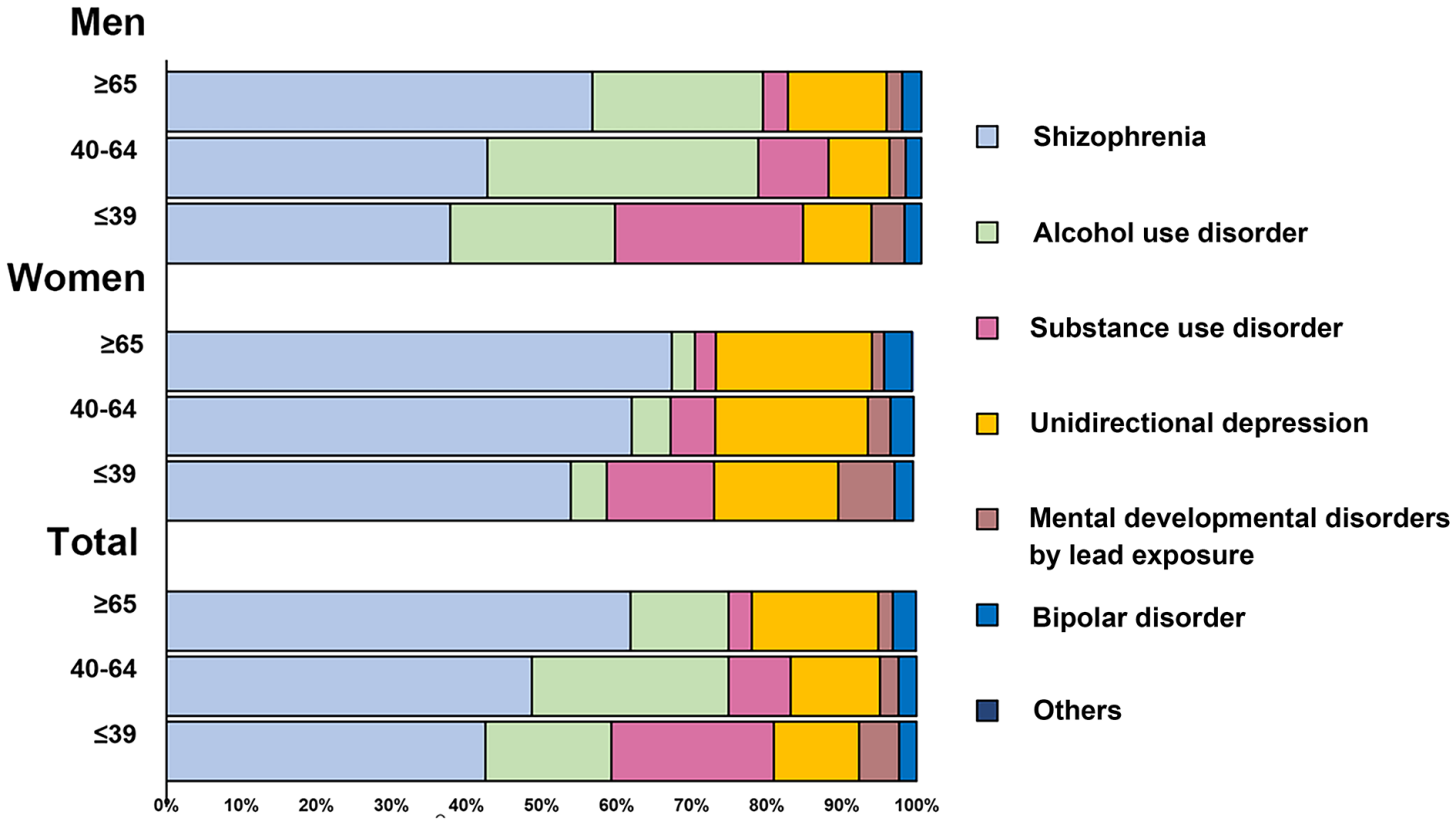
Proportion of age-standardized mortality rates per 100,000 persons for the major causes of mental disorder in China, 2009–2019.

The ASMRs of schizophrenia, AUD, unidirectional depression, and bipolar disorder were highest in the 40–64 years old group, while SUD and other mental developmental disorders were higher in the age ≤ 39 years old group than in other age groups (Supplementary Figure S2). In the whole population, schizophrenia was the leading cause of death associated with MD in every age group (Figure 3). The proportion of deaths from schizophrenia and unidirectional depression increased with the increase in age. In men, under 39 years of age, schizophrenia (54.09%), SUD (24.99%), and AUD (21.85%) were the top 3 causes of MD death; in the age group of 40–64 years, schizophrenia accounted for 42.42% of all MD deaths, followed by AUD (35.87%) and SUD (9.36%); in men over 60 years of old, 56.37% of MD deaths were schizophrenic, 22.62% were AUD, and 13.01% were unidirectional depression. In women, under 39 years of age who died of MD, schizophrenia accounted for 54.09%, followed by depression (16.56%) and unidirectional depression (14.35%); in women aged 40–64 years, schizophrenia accounted for 62.27% of MD deaths, followed by unidirectional depression (24.99%), and SUD (21.85%); in women over 60 years old, the first cause of MD deaths was schizophrenia (67.59%), followed by unidirectional depression (20.87%).

### Geographic characteristics analysis of the major MD

The ASMR for all types of MD was found to be highest in the western regions, followed by the eastern regions, and the lowest in the central regions. Furthermore, the urban–rural disparity increased from the central to eastern to western regions, as shown in Table 1. The ASMR for all types of MDs was higher in men than in women. It was observed that the ASMR for all types of MD was lower in urban residents as compared to rural residents, with the highest ASMR observed in the age group of 40–64 years (Table 1). The risk of MD in eastern rural residents was lower than that in eastern urban residents (RR = 0.825, 95%Cl 0.755–0.901, *p* < 0.001). Meanwhile, rural residents in the west had a 31% higher risk of MD than urban residents in the west (RR = 1.310, 95% CI 1.195–1.435, *p* < 0.001).

**Table 1:** Rate ratio of mental disorder mortality between urban and rural residents by region, sex, and age in China, 2009–2019.

		Urban		Rural			
Subgroup		Total people	Mortality rate (per 100,000)	Total people	Mortality rate (per 100,000)	RR (95% CI)	*p*-value
Region	Eastern	2,642	0.57	3,873	0.51	0.825 (0.755–0.901)	<0.001
	Central	1,350	0.44	3,382	0.47	0.987 (0.901–1.083)	0.789
	Western	1777	0.68	5,154	1.11	1.310 (1.195–1.435)	<0.001
Sex	Male	3,464	0.83	7,897	0.68	1.079 (0.986–1.181)	0.096
	Female	2,305	0.46	4,512	0.44	0.927 (0.847–1.014)	0.096
Age	≤39	1,060	0.18	2,505	0.13	0.956 (0.703–1.301)	0.771
	40–64	3,032	0.31	6,334	0.28	0.928 (0.660–1.305)	0.660
	≥65	1,677	0.16	2,570	0.15	1.036 (0.582–1.844)	0.904
Overall		5,768	0.56	12,409	0.65		

**Figure 4:**
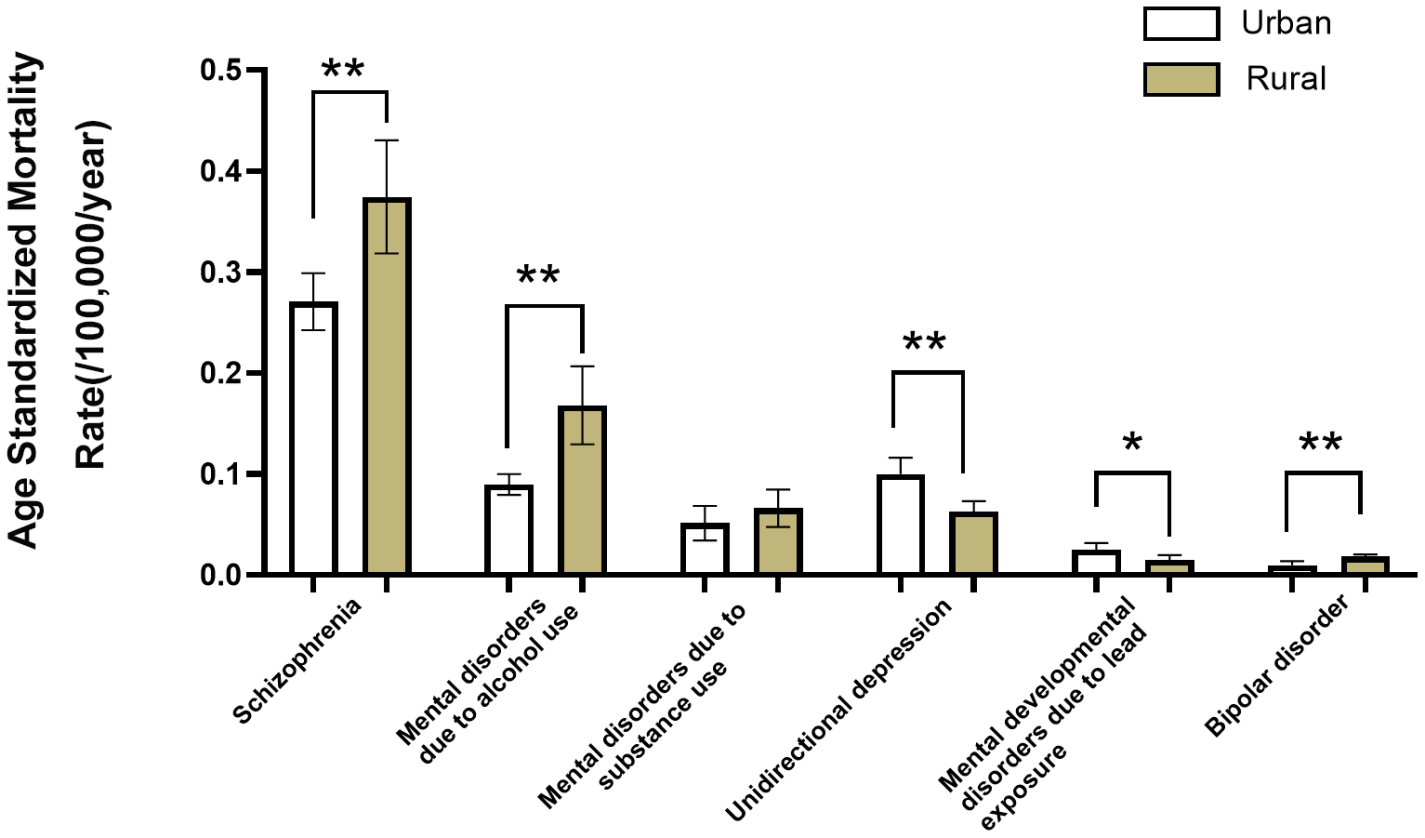
The age-standardized mortality for main mental disorder types in urban and rural populations in mainland China during 2009–2019. Comparison of the age-standardized mortality rates of the major mental disorder types in urban and rural populations.

**Figure 5:**
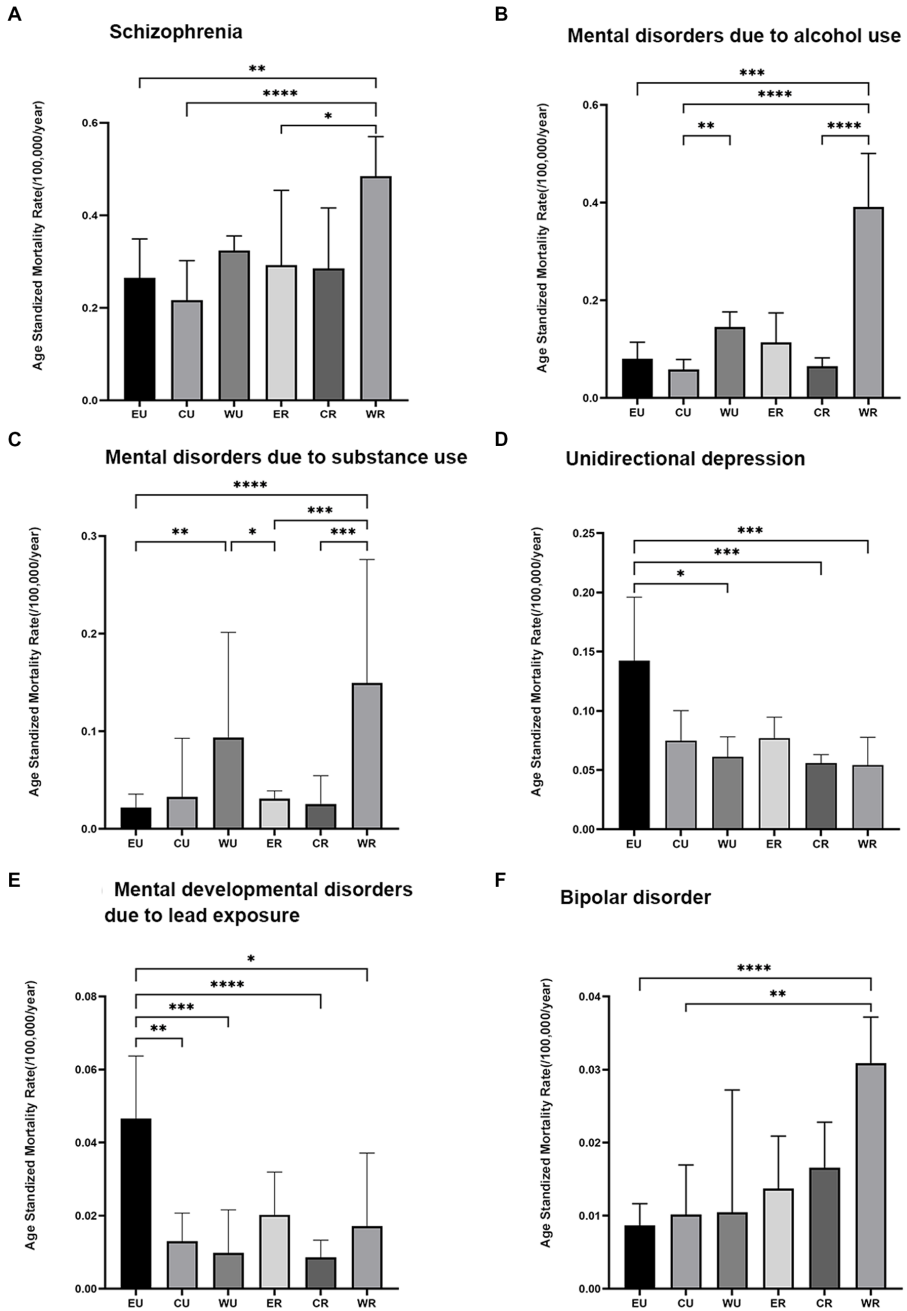
Trends in the age-standardized mortality rates of the major mental disorder types in the eastern, central, and western regions of mainland China during 2009–2019. EU = eastern rural area, CU = central rural area, WU = western rural area, ER = eastern urban area, CR = central urban area, WR = western urban area, * indicates *p* < 0.05, ** indicates *p* < 0.01, *** indicates *p* < 0.001, **** indicates *p* < 0.0001. The Bonferroni correction has adjusted significance values for multiple tests. **A**: Schizophrenia; **B**: Mental disorders due to alcohol use; **C**: Mental disorders due to substance use; **D**: Unidirectional depression; **E**: Mental developmental disorders due to lead exposure; **F**: Bipolar disorder.

Among the six major MDs, AUD had a higher ASMR in rural residents compared to urban residents (*p* < 0.001). Unipolar depression and lead exposure-induced mental developmental disorders had significantly higher ASMRs in urban residents compared to rural residents (*p* = 0.001 and *p* = 0.012, respectively; Figure 4). The ASMR for schizophrenia (Figure 5A), AUD (Figure 5B), SUD (Figure 5C), and bipolar disorder (Figure 5F) was highest in the western rural areas, while the eastern urban areas had the highest ASMR for unipolar depression (Figure 5D) and developmental MD caused by lead exposure (Figure 5E). Furthermore, the ASMR of AUD was lowest in the central regions, followed by the eastern regions and finally the western regions (Figure 5B). The ASMR for SUD in western regions was significantly higher than that in eastern regions (Figure 5C).

### Burden of the major MD in China, 2009–2019

**Figure 6:**
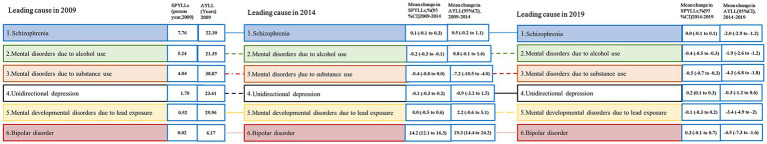
Changes in the age-standardized mortality for main mental disorder in China, 2009–2019. Solid lines are “increases” and dashed lines are “decreases”. For the time period 2009–2014 and 2014–2019, two measures of change are shown: percent change in SPYLLs, change in SPYLLs, change in AYLL. Statistically significant changes are shown with *. SPYLLs = age-standardized person years of life lost per 100,000 persons. AYLL = average years of life lost.

Schizophrenia and AUD were consistently identified as the top two causes of MD-related deaths throughout the study period (Figure 6). The SPYLLs and AYLLs of schizophrenia were 7.76 person-years and 22.30 years in 2009, respectively, and remained stable for 11 years.

The SPYLLs of AUD decreased by 0.2% (95% CI 0.3 to 0.1), while those caused by SUD decreased by 0.4% (95% CI −0.8 to 0.0), and the AYLLs caused by SUD decreased by 7.29 (95% CI −10.5 to −4.0). The SPYLLs of bipolar disorder increased by 14.2% (95% CI 12.1 to 16.3) during this period. From 2014 to 2019, the SPYLLs of AUD decreased by 0.4% (95% CI −0.5 to −0.3), the SPYLLs of SUD decreased by 0.5% (95% CI −0.7 to 0.3) and the AYLL decreased by 1.9 (95% CI −2.6 to −1.2), while the SPYLLs of unidirectional depression increased by 0.2 (95% CI 0.1 to 0.3).

## Discussion

In this study, the overall profile and trends of MD deaths in mainland China during 2009–2019 were evaluated. The ASMR for MD in China was 0.62 deaths per 100,000 per year, representing 0.13% of all deaths. Although a mortality rate of 0.13% is low, given China’s huge population of 1.4 billion, the absolute number of individuals dying from MD remains significant. The rankings of the three leading causes of MD death in the annual reports were schizophrenia, AUD, and SUD between 2009 and 2014, while unidirectional depression surpassed SUD in the year of 2015, and became one of the top three causes of MD death from 2015 to 2019. Overall, the ASMR of MD was higher in men than in women, but did not vary significantly between urban and rural residents. The CMR of all MDs remained stable while their ASMR decreased, indicating that aging partly contributed to the increase in MD deaths.

As the leading cause of MD deaths in all age groups, schizophrenia accounted for 50.7% of MD death in this study. Schizophrenia is associated with a 5% risk (22) of suicide and other negative outcomes such as cognitive impairment, inflammation, and oxidative stress, which worsen with age in people with schizophrenia. The reason remains unclear. Schizophrenia patients have a mortality rate that is more than twice that of the general population (1). Moreover, they also have a higher suicide rate compared to the general population (2).The methylation status of aging individuals with schizophrenia was identified as being at risk for suicide. Therefore, high-throughput investigations of peripheral blood methylation status can be used as biomarkers to identify patients with schizophrenia who are at risk of suicide. Through tailored treatment, and ultimately, to prevent suicide in this population (22). The elevated mortality rate observed in individuals with mental disorders can be attributed to several factors. People with schizophrenia may have inadequate access to healthcare and limited self-management skills, which can result in an unhealthy lifestyle. Furthermore, certain antipsychotic medications may increase the risk of cardiovascular disease and other conditions, further raising the risk of death in these patients (3).

In contrast to other MD types, SUD was more prevalent in the group age ≤ 39 years old than in other age groups, which may be linked to the high incidence of MD and SUD among adolescents and young adults and the increased likelihood of co-occurrence (23). It has been proven that in the United States, about 41–65% of substance abusers suffer from MD. More than 90% of adults with SUD start using alcohol or drugs during adolescence (24–26). Individuals with substance abuse disorders within the last 12 months were found to be 4.2 times more likely to have co-occurring MD than non-substance abusers, and 2.9 times more likely than non-addicts (27). Furthermore, the ASMR of AUD was observed to be higher in individuals aged 40–64 years compared to other age groups. This trend may be attributed to the fact that males who are between the ages of 45–54 and consume alcohol have the highest rates of AUD (28). Binge drinking is common in China for social and business reasons (29), and drinkers may not stop drinking until they retire (age 60) or their physical health deteriorates significantly. The excess mortality observed in individuals with AUD can be attributed to both the predisposing factors that led to the development of AUD (which may also be shared by close family members) and the direct effects of AUD itself (30).

This study also demonstrated that the ASMRs of schizophrenia, AUD, and bipolar disorder were higher in rural residents than in urban residents. This could be attributed to the fact that people with schizophrenia often experience conduct disorders, which increases the risk of unnatural deaths such as suicide, homicide, and accidents (31). Additionally, differences in access to quality healthcare, social determinants of health, and lifestyle factors may also contribute to the disparities in mortality rates between rural and urban residents with MD. The prevalence of schizophrenia is positively correlated with urbanization level (32, 33), and the higher mortality rate of schizophrenia in rural areas may be due to the lower access to and limited capacity of emergency medical services in rural areas compared with urban areas, leading to higher unnatural mortality (34). The higher western rural prevalence suggested that schizophrenia is more prevalent among people of lower socioeconomic status tend to be in poorer health status (35) associated with suicide, accidental death, and injury (36). In developing countries, such as China, treatment rates for schizophrenia are still relatively low (37). A survey found that untreated status (46.3%) and poor treatment status were prominent problems among elderly patients with severe MD in rural China (38). Mental health services in rural areas were poorer than urban areas in China and a lack of medical knowledge may affect attitudes toward medication among individuals and their families, which may lead to higher mortality rates. There are similar reasons for the high rural mortality rates of AUD and bipolar disorder. Rural residents are also more likely than urban residents to develop alcohol dependence (7). Regional variations in AUD mortality are also associated with different drinking practices across regions, and strategies to change collective culturally and socially acceptable alcohol drinking patterns can reduce the burden of alcohol-related disease (39). Effective interventions should be explored with the aim of preventing alcohol abuse.

Unipolar depression was identified as one of the leading causes of MD-related deaths in women, accounting for 19.1% of all MD deaths. According to a previous study, the estimated prevalence rates of major depressive disorder in China were 1.6, 2.3, and 3.3% for current, 12-months, and lifetime prevalence, respectively (40). Additionally, depression was responsible for over 10 million disability-adjusted life years (DALYs) lost in China in 2013, and this number was projected to increase by approximately 10% by 2025 (41). In this study, there was a 25.4% increase in 2019 compared to SPYLLs in 2009. Areas with a high level of economic development also mean greater pressure from work and life. Women and those in the 40–65 age group are at high risk of unidirectional depressive death. Several studies have manifested gender differences in depression (42–44), and it is now widely believed that women are more likely to experience negative sexual events (45), leading to more negative emotions. Depression is a very costly disorder in China (46), and how to popularize social support and psychological treatment is a problem that needs to be considered.

The ASMR of MD was the highest in the western regions with poor topographic conditions and dispersed industrial layout, followed by the eastern regions with developed economies and high living pressure, and finally the central regions, which reflected the regional distribution characteristics. In the vast western regions, the ASMR of MD in rural residents is higher than in urban residents. On the contrary, in the economically advanced eastern regions, the mortality rate of MD in urban residents is higher than that in rural residents. Differences in mortality among people with MD are likely influenced by a multitude of factors, such as behavioral and lifestyle choices, quality and availability of healthcare, as well as social determinants of health, including poverty and social connectedness (47, 48). Targeted interventions must take into account national and local political and economic environments and require a significant reallocation of social and health resources to address a problem of this scale.

In addition to suicide, a significant portion of the increased mortality in patients with mental disorders can be attributed to physical illnesses (1), which may be related to the use of antipsychotic medications. Psychotropic drugs can further raise the risk of metabolic abnormalities in these patients (2). Weight gain is a well-established side effect of antipsychotics (3). In addition to complications arising from weight gain and obesity-related mechanisms, antipsychotics can directly impact cardiovascular risk. Certain antipsychotic drug metabolites can be toxic, such as chlorpromazine metabolites which can cause arrhythmia, heart failure, and other adverse reactions leading to patient death (4).The duration of mental disorders, age of onset, number of hospitalizations, and other factors may also influence patient mortality. Long-term mental illness can result in various physical and psychological problems, such as poor nutrition, cardiovascular disease, and drug side effects, which can increase the risk of death. In contrast, patients with a short course of illness may be more easily cured or recover faster, resulting in a lower mortality rate. The age of onset of mental illness can also affect mortality. Some mental illnesses, like schizophrenia and bipolar disorder, often develop at a young age and can affect patients’ lives and careers, increasing their risk of death if not treated promptly and effectively (48). On the other hand, some elderly people may suffer from mental illnesses due to loneliness, physical degradation, and other factors, which can increase their mortality rate. The number of times a patient is hospitalized can also impact mortality. Depending on the severity of the mental illness, hospitalization frequency can vary. Patients may face an increased risk of death every time they are hospitalized, especially if the mental illness is severe (49). Other factors, such as treatment effectiveness, social support network, and physical condition, may also affect patient mortality. Poor treatment, lack of social support, or poor physical condition can increase the risk of death (2).

This study has several limitations that should be acknowledged. Firstly, it only considers deaths that are clearly classified as mental and substance use diseases, which may lead to an underestimation of mortality. Secondly, the ICD guidelines require the recorded cause of death to be the primary or direct cause of death, which may mask the contribution of other underlying causes of death. Meanwhile, Global Burden Disease (GBD) 2010 estimated that most of the disease burden caused by mental, neurological, and substance use disorders is due to non-fatal health losses, which were not calculated in this study due to lack of data on the prevalence of mental disorders in the population; only 15% of the total burden comes from mortality in years of life lost (YLLs) (50). MD is a multi-family chronic disease with a long course and low mortality. It is underestimated to evaluate the impact of mental disorders on population health only by mortality (51). Additionally, changes in the number of national disease detection points in 2013 may have had an impact on the statistical results of disease mortality. Population coverage may differ between data sources before and after 2013, which could affect the accuracy of the data. Therefore, it is important to consider the impact of changes in national disease detection points when interpreting the results of disease mortality statistics.

## Conclusion

Our findings not only reflect the natural course of MD death but also highlight the need for targeted efforts to strengthen regional mental health weaknesses. Preventive strategies focusing on schizophrenia should be considered by governments to decrease MD-associated deaths. In western China, with an underdeveloped economy, more treatment and assistance policies were suggested to take steps to help patients with MD. Health education focus on substance abuse and behavioral intervention should be taken into account among the young population under 40 years old. In addition, the social welfare department can provide a livelihood position for MD patients whose symptom control is stable and their working ability is acceptable.

## Data availability statement

The original contributions presented in the study are included in the article/Supplementary materials, further inquiries can be directed to the corresponding authors.

## Author contributions

JW and LZ had full access to all the data in the study and took responsibility for the integrity of the data and the accuracy of the data analysis. LZ and JL: concept, design, and supervision. JW, YW, LW, and HW: acquisition, analysis, or interpretation of data. JW and LZ: drafting of the manuscript. LZ, JW, YW, LW, HW, and JL: critical revision of the manuscript for important intellectual content. JW: statistical analysis. LZ: obtained funding, administrative, technical, or material support. All authors contributed to the article and approved the submitted version.

## Funding

This work was supported by the National Natural Science Foundation of China (Grant No. 81872720) and Shanghai Municipal Health Commission (Grant No. 201840066) to LZ.

## Conflict of interest

The authors declare that the research was conducted in the absence of any commercial or financial relationships that could be construed as a potential conflict of interest.

## Publisher’s note

All claims expressed in this article are solely those of the authors and do not necessarily represent those of their affiliated organizations, or those of the publisher, the editors and the reviewers. Any product that may be evaluated in this article, or claim that may be made by its manufacturer, is not guaranteed or endorsed by the publisher.

## Supplementary material

The Supplementary material for this article can be found online at: https://www.frontiersin.org/articles/10.3389/fpsyt.2023.1169502/full#supplementary-material
