# Supplementary material for: Trends and burden in mental disorder death in China from 2009 to 2019: a nationwide longitudinal study
Source: Front Psychiatry. 2023 May 12;14:1169502. doi: 10.3389/fpsyt.2023.1169502 (PMC10274321; doi:10.3389/fpsyt.2023.1169502)
Supplement: Supplementary file 1 [file Data_Sheet_1.docx]

**Supplementary Material**

**Journal name:** **Social psychiatry and psychiatric epidemiology**

**Trends and burden in mental disorder death in China from 2009 to 2019: a nationwide longitudinal study**

Jiawen Wu^1,2^; Yuzhu Wang^1,2^; Lu Wang^1,2^; Hengjing Wu^1^, Jue Li^1,2*^; Lijuan Zhang^1,2*^

^1^Clinical Center for Intelligent Rehabilitation Research, Shanghai YangZhi Rehabilitation Hospital (Shanghai Sunshine Rehabilitation Center), Tongji University School of Medicine, Tongji University, Shanghai, 201613, China;

^2^Department of Epidemiology, Tongji University School of Medicine, Shanghai, 200092, China;

*Correspondence: Jue Li (jueli@tongji.edu.cn) & Lijuan Zhang ([zhangxiaoyi@tongji.edu.cn](mailto:zhangxiaoyi@tongji.edu.cn))

**
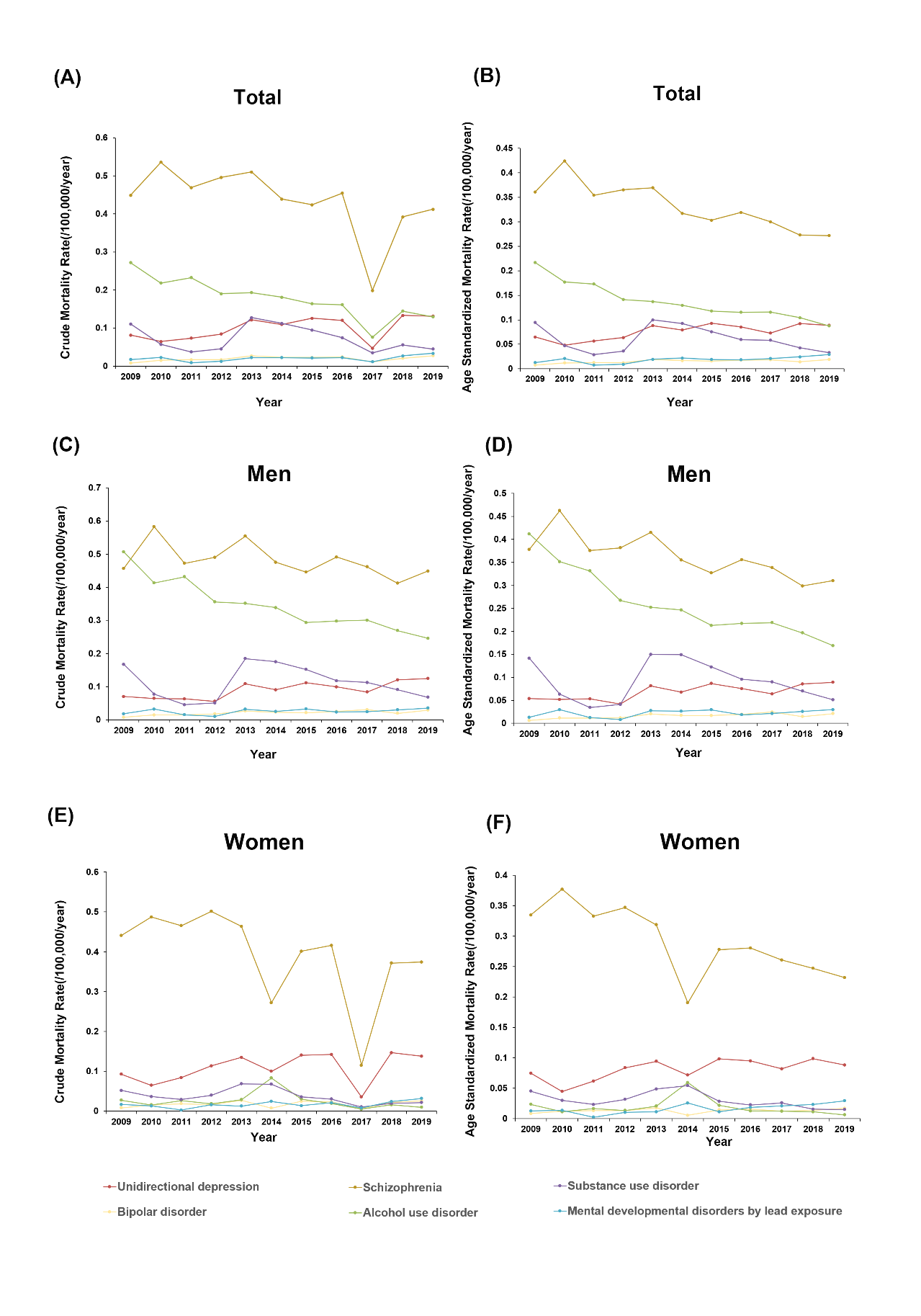
**

**Figure S1.** Trends in crude and age-standardized mortality rates of the major causes of mental disorder in both sexes in China, 2009–2019.

(A) trend in crude mortality rates in the total population

(B) trends in age-standardized mortality rates in the total population

(C) trend in crude mortality rates in men

(D) trends in age-standardized mortality rates in men

(E) trends in crude mortality rates in women

(F) trend in age-standardized mortality rates in women


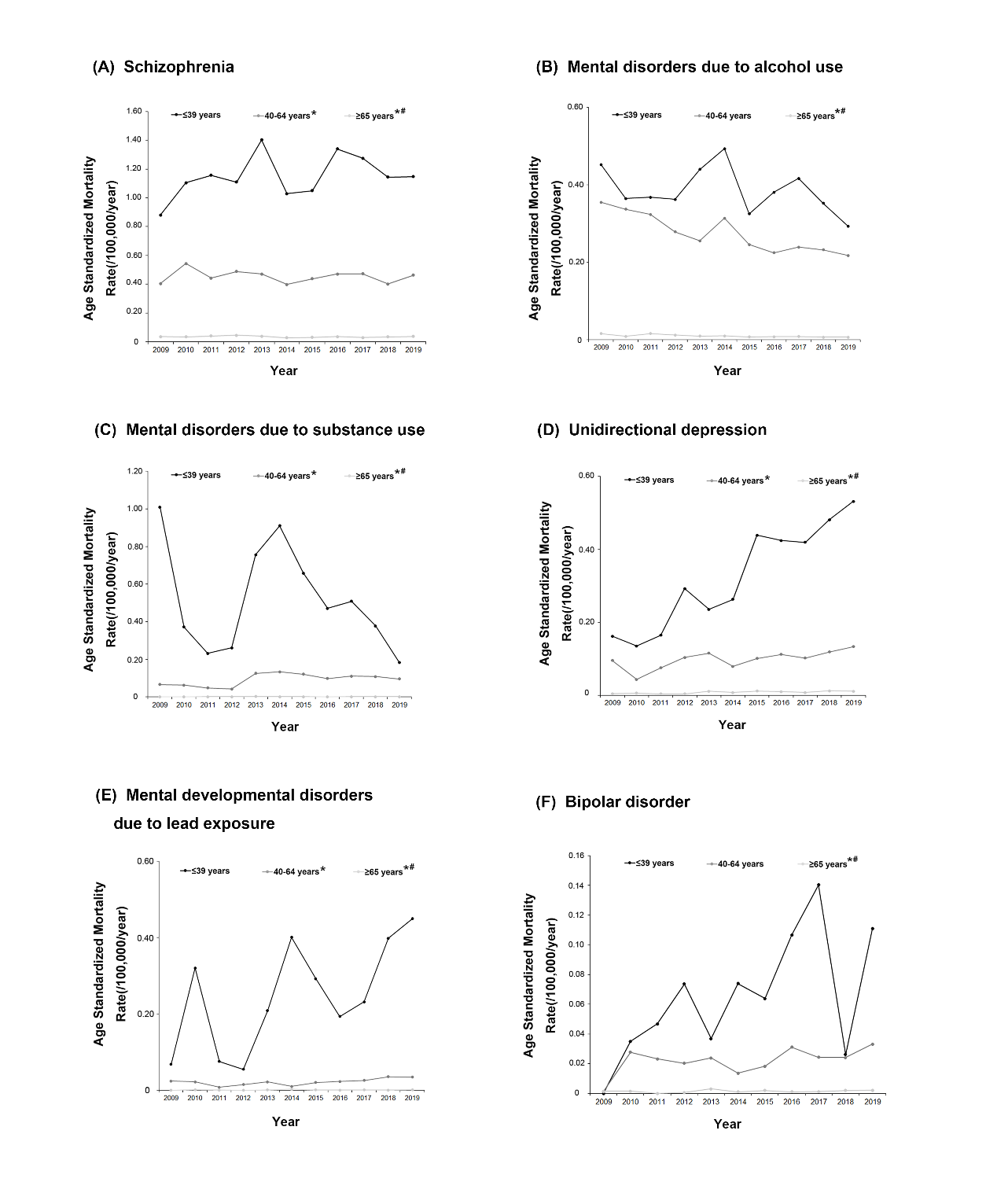


**Figure S2.** Trends in the age-standardized mortality for main mental disorder types in three age groups (≤39 years, 40-64 years, ≥65 years) in China, 2009–2019

Note: * indicates compared to ≤39 years group, *P*<0.05; # indicates compared to 40-64 years group, *P* <0.05

| **Table S1.** List of International Classification of Diseases (ICD) codes mapped to the Global Burden of Disease cause list for causes of mental disorder | |
| --- | --- |
| **Cause** | **ICD-10** |
| Mental disorders | F01-F099 |
| Unidirectional depression | F32-F33 |
| Bipolar disorder | F30-F31 |
| Schizophrenia | F20-F29 |
| Mental disorders due to alcohol use | F10 |
| Mental disorders due to substance use | F11-F16,F18-F19 |
| Post-traumatic stress disorder | F43.1 |
| Obsessive compulsive disorder | F42 |
| Panic disorder | F40.0 |
| Phobia | F41.0 |
| Insomnia | F51 |
| Mental developmental disorders due to lead exposure | F70-F79 |

**Table S1.** List of International Classification of Diseases (ICD) codes mapped to the Global Burden of Disease cause list for causes of mental disorder

**Table S2.** Total number of persons surveyed, deaths and deaths with mental disorders in Urban and rural China, 2009-2019

**Table S3.** Age standardized mortality rates (100, 000 per year) of Mental disorder by gender, urban/rural residence and type in China, 2009–2019.

**Table S4.** Trends in ASMR for MD by gender, urban/rural residence and age group,2009-2019

**Table S5.** Trends in mortality rate for the major types of MD by gender and urban/ rural China,2009-2019

**Table S6.** The five leading causes of mental disorder mortality rates (95%CI) per 100, 000 among urban and rural residents by region, sex and age in China, 2009–2019
